# Using Intervention Mapping and Behavior Change Techniques to Develop a Digital Intervention for Self-Management in Stroke: Development Study

**DOI:** 10.2196/45099

**Published:** 2023-07-24

**Authors:** Alex W K Wong, Mandy W M Fong, Elizabeth G S Munsell, Christopher L Metts, Sunghoon I Lee, Ginger E Nicol, Olivia DePaul, Stephanie E Tomazin, Katherine J Kaufman, David C Mohr

**Affiliations:** 1 Center for Rehabilitation Outcomes Research Shirley Ryan AbilityLab Chicago, IL United States; 2 Department of Physical Medicine and Rehabilitation, Northwestern University Feinberg School of Medicine Chicago, IL United States; 3 Department of Medical Social Sciences, Northwestern University Feinberg School of Medicine Chicago, IL United States; 4 Center for Education in Health Sciences, Feinberg School of Medicine Chicago, IL United States; 5 Department of Pathology and Laboratory Medicine, Medical University of South Carolina Charleston, SC United States; 6 Manning College of Information and Computer Sciences, University of Massachusetts Amherst Amherst, MA United States; 7 Department of Psychiatry, Washington Univesity School of Medicine St. Louis, MO United States; 8 Memorial Hospital Belleville, Barnes-Jewish/Christian HealthCare Belleville, IL United States; 9 Center for Behavioral Intervention Technologies, Northwestern University Feinberg School of Medicine Chicago, IL United States; 10 Department of Preventive Medicine, Northwestern University Feinberg School of Medicine Chicago, IL United States

**Keywords:** mobile health, digital intervention, technology, SMS text messaging, intervention mapping, behavior change, self-management, stroke, rehabilitation, mobile phone

## Abstract

**Background:**

Digital therapeutics, such as interventions provided via smartphones or the internet, have been proposed as promising solutions to support self-management in persons with chronic conditions. However, the evidence supporting self-management interventions through technology in stroke is scarce, and the intervention development processes are often not well described, creating challenges in explaining why and how the intervention would work.

**Objective:**

This study describes a specific use case of using intervention mapping (IM) and the taxonomy of behavior change techniques (BCTs) in designing a digital intervention to manage chronic symptoms and support daily life participation in people after stroke. IM is an implementation science framework used to bridge the gap between theories and practice to ensure that the intervention can be implemented in real-world settings. The taxonomy of BCTs consists of a set of active ingredients designed to change self-management behaviors.

**Methods:**

We used the first 4 steps of the IM process to develop a technology-supported self-management intervention, interactive Self-Management Augmented by Rehabilitation Technologies (iSMART), adapted from a face-to-face stroke-focused psychoeducation program. Planning group members were involved in adapting the intervention. They also completed 3 implementation measures to assess the acceptability, appropriateness, and feasibility of iSMART.

**Results:**

In step 1, we completed a needs assessment consisting of assembling a planning group to codevelop the intervention, conducting telephone surveys of people after stroke (n=125) to identify service needs, and performing a systematic review of randomized controlled trials to examine evidence of the effectiveness of digital self-management interventions to improve patient outcomes. We identified activity scheduling, symptom management, stroke prevention, access to care resources, and cognitive enhancement training as key service needs after a stroke. The review suggested that digital self-management interventions, especially those using cognitive behavioral theory, effectively reduce depression, anxiety, and fatigue and enhance self-efficacy in neurological disorders. Step 2 identified key determinants, objectives, and strategies for self-management in iSMART, including knowledge, behavioral regulation, skills, self-efficacy, motivation, negative and positive affect, and social and environmental support. In step 3, we generated the intervention components underpinned by appropriate BCTs. In step 4, we developed iSMART with the planning group members. Especially, iSMART simplified the original psychoeducation program and added 2 new components: SMS text messaging and behavioral coaching, intending to increase the uptake by people after stroke. iSMART was found to be acceptable (mean score 4.63, SD 0.38 out of 5), appropriate (mean score 4.63, SD 0.38 out of 5), and feasible (mean score 4.58, SD 0.34 out of 5).

**Conclusions:**

We describe a detailed example of using IM and the taxonomy of BCTs for designing and developing a digital intervention to support people after stroke in managing chronic symptoms and maintaining active participation in daily life.

## Introduction

### Background

Stroke is the leading cause of long-term disability in the United States. The American Heart Association estimates that 795,000 individuals experience a new or recurrent stroke each year in the United States, and the annual cost of stroke is US $45.5 billion [1]. Globally, the prevalence of stroke is projected to affect 77 million people by 2030 owing to reducing stroke mortality rates and an ever-aging population [2]. Many people live with residual disabilities after stroke, such as reduced mobility and limitations in performing household chores and community activities [3]. Stroke often manifests uncontrolled chronic conditions (eg, diabetes and hypertension), and people after stroke also experience ongoing chronic symptoms (eg, depression and fatigue). The inability to manage chronic conditions and symptoms may lead to decreased participation in prestroke roles and activities, exacerbating other chronic diseases and increasing the chance of stroke recurrence and mortality [3,4]. Moreover, the impact of stroke on personal health and health care will continue to grow, forcing an urgent demand for innovative person-centered approaches in stroke care [5].

In stroke rehabilitation, an effective approach to addressing these long-term consequences is teaching self-management skills for people after stroke [5,6]. Self-management is defined as an individual’s behavioral management of symptoms, treatment, physical and psychosocial consequences, and lifestyle changes inherent in living with a chronic disease [7]. Self-management can be delivered in various formats; the most common delivery format is via group-based face-to-face structured education programs [8]. A meta-review of 13 systematic reviews has demonstrated that self-management interventions improve daily activities, independence, and mortality in people after stroke [9]. National research agendas, such as those of the Department of Health and Human Services [10] and the Institute of Medicine [11], include self-management as a key strategic framework for promoting health, managing chronic conditions, and preventing disability.

Although self-management programs are recommended, evidence has shown that the uptake of these structured education programs is low [12,13]. Work obligations, time commitments, and low perceived benefits are commonly identified barriers contributing to the low uptake of these programs [12]. Fortunately, growing evidence supports using technologies for self-management and other behavior change interventions [14]. Digital interventions that use a broad range of technologies, such as mobile phones, the web, and sensors, have become a new means of delivering care and offering support for users in changing behaviors and cognitions related to health, mental health, and wellness [15]. A systematic review of reviews indicates that SMS text messaging interventions effectively support self-management of chronic diseases because of their high reach and highly accessible, relatively low-cost communication strategy [16]. Moreover, the American Heart Association states that digital interventions have the potential to revolutionize self-management by promoting patient and clinician engagement in active real-time care partnerships [17]. Indeed, some barriers associated with face-to-face programs, such as transportation, location, and time, are easier to address with digital interventions because users can access digital interventions at their own time and place [18].

Despite digital interventions for self-management becoming more available, the descriptions of the intervention development process are often unclear [19] and do not consistently incorporate both the patient and clinician perspectives; moreover, none exist for people after stroke. The findings of a recent systematic review regarding digital self-management interventions in low back pain have supported similar arguments. The authors found that the articles included in the review were heterogeneous and did not report intervention details, making it difficult to understand what might work best, for whom, and in what circumstances [20]. Intervention mapping (IM) provides a well-defined framework for systematically developing, implementing, and evaluating behavior change interventions [21,22]. IM emphasizes the participation of stakeholders and provides a structure for integrating theory, findings from empirical literature, and information collected from the target populations. The IM approach has been applied in designing digital self-management interventions in other chronic conditions, such as type 2 diabetes [18] and low back pain [23], as well as nondigital self-management programs [24-26].

### Objectives

This paper provides a use case of the step-by-step IM process used to adapt an evidence-based face-to-face self-management education program, Improving Participation After Stroke Self-Management (IPASS), which consists primarily of group-based psychoeducation [27], into an interactive Self-Management Augmented by Rehabilitation Technologies (iSMART) intervention delivered via a mobile device. We chose to adapt IPASS, which is a stroke-specific self-management intervention targeting the development and practice of chronic disease management skills to improve functioning and participation in home, work, community, and social activities. Digital tools such as iSMART can address the common barriers to participation in traditional face-to-face self-management programs, such as time, transportation, or geographic location, that limit access to, and uptake of, self-management behaviors. To improve engagement in self-management activities among people after stroke, we added a 12-week interactive SMS text messaging component to iSMART to identify and set treatment goals and provide daily goal-specific tips, personalized reminders, and motivational messaging, as well as a weekly goal check-in to promote the self-monitoring of progress. As people after stroke often experience restricted life participation, we also incorporated weekly coaching sessions into the existing iSMART framework to incorporate live weekly health coaching sessions to assess progress toward goals as well as identify barriers to engagement in self-management behaviors and strategies to address barriers, including changing or adjusting goal type or difficulty.

We hypothesized that engaging clinicians and researchers in the iSMART development and adaptation process using the IM framework would benefit the development process by incorporating the perspective of real-world clinical practice and improving the understanding of technology-supported self-management approaches, thereby increasing the uptake of such tools in the clinical management of stroke recovery. Here, we describe the use of IM to systematically adapt evidence-based self-management approaches for digital delivery, incorporating the perspectives of patients, their caregivers, and clinicians who treat people after stroke. The process and basic intervention framework may be relevant to other chronic conditions where self-management is key to promoting recovery and maintaining functional gains achieved during recovery.

## Methods

### Overview of iSMART’s Intervention Architecture and Theoretical Foundations

We first present an overview of iSMART and its theoretical foundations so that readers can better understand iSMART intervention. Behavioral determinants, mechanisms of action (MoAs), and screenshot examples are presented alongside the IM processes described later. iSMART is a 12-week digital intervention to improve skills to manage chronic conditions and support daily activity participation for people after stroke. iSMART consists of 3 components: a group psychoeducation program for skills training and practices, individual coaching, and SMS text messaging. The psychoeducation component of iSMART, which was adapted from IPASS, combines two theoretical approaches: (1) a chronic disease self-management program built on social cognitive or learning theory [28] and (2) community participation and environment management built on the person-environment-occupation-performance model [29]. iSMART’s psychoeducation component focuses on teaching 5 self-management skills (ie, problem-solving, accommodations, communication, decision-making, and emotion and symptom management) to manage chronic conditions and support daily activity participation in people after stroke.

The original IPASS program did not include a coaching component. Live health coaching is effective in chronic disease management and as such is commonly used across several chronic disease states as a tool for both treatment and prevention. Health coaching was incorporated into iSMART to promote collaborative goal setting and provides a mechanism for discussing and adjusting goal type and difficulty to promote self-efficacy for self-management behaviors. The coaching component of iSMART was grounded in principles of behavioral activation [30]. We adapted the Brief Behavioral Activation Treatment for Depression-Revised [31] manual for iSMART, in particular adapting the methods for identifying treatment goals based on valued life areas and activities, mechanisms for monitoring progress, and potential barriers to engagement or success, as well as proactively developing strategies to achieve goals based on these inputs. Both psychoeducation and coaching sessions were delivered weekly via videoconferencing for the first 7 weeks. Table 1 shows the content of the psychoeducation and coaching sessions.

**Figure 1:**
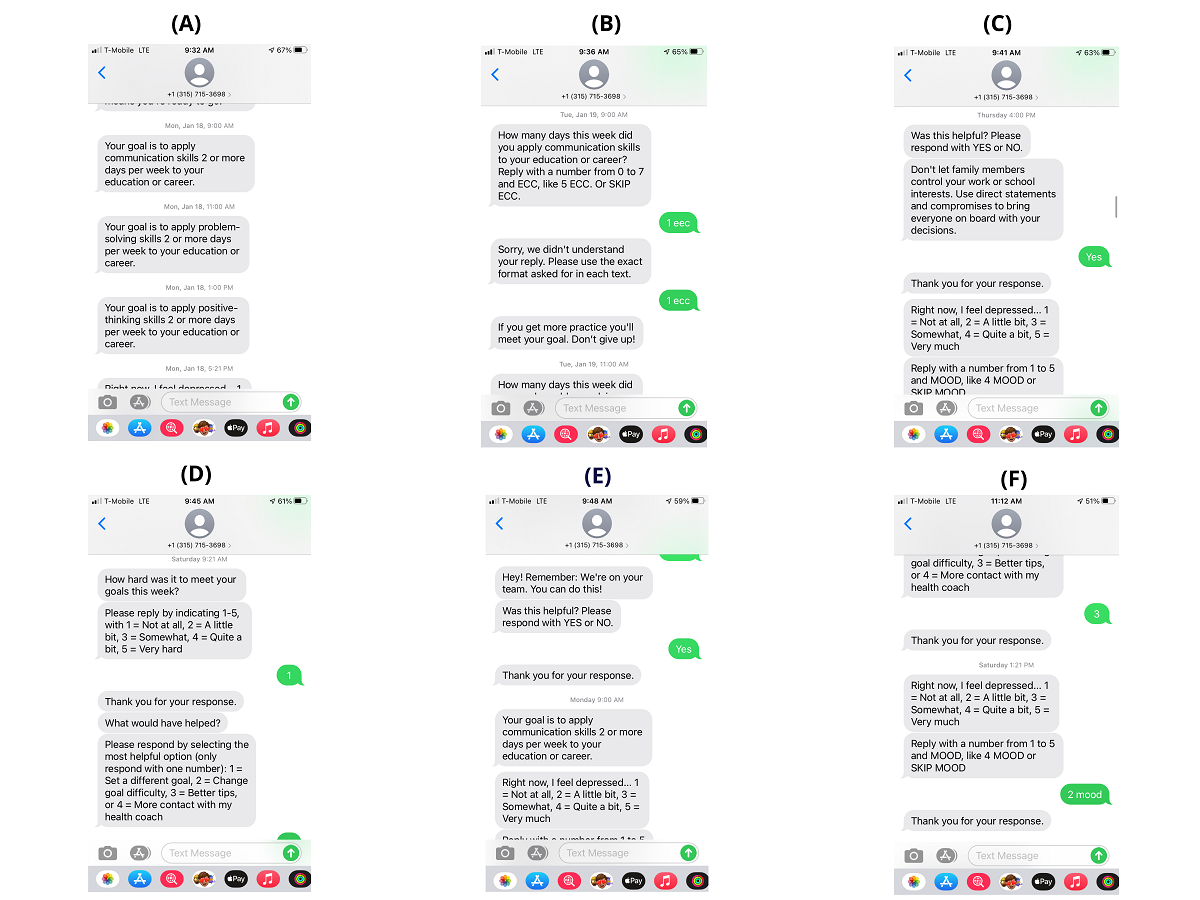
Screenshots of various types of SMS text messages. (A) goal reminder; (B): goal check-in; (C) self-management tip; (D) ecological needs assessment; (E) general motivation; and (F) mood check-in.

**Figure 2:**
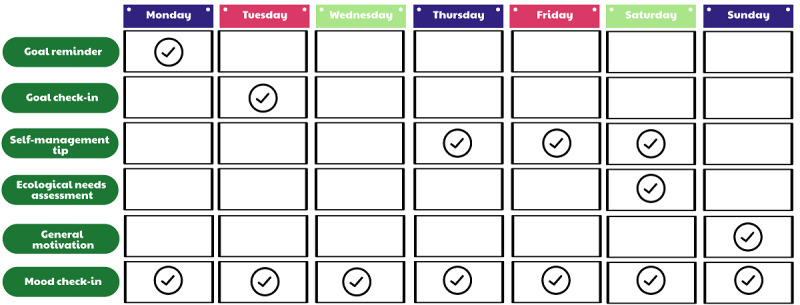
SMS text messaging schedule.

To improve user engagement, we added the SMS text messaging component to iSMART to further support self-management behaviors. The messaging framework used in this study was adapted from prior studies with effectiveness demonstrated in hospital workers [32,33] and adults with severe mental illness [34]. As a first step toward adapting the platform for improving engagement in, and uptake of, self-management behaviors in people after stroke, content was adapted. Specifically, SMS text messaging content for goal reminders, self-management tips, motivational messaging, and monitoring for psychological distress (such as depressed mood) was customized to meet the needs of people after stroke and incorporated into the existing SMS text messaging framework. Figure 1 shows screenshots of different types of SMS text messages. These messages were sent following a predefined weekly schedule (Figure 2).

**Table 1:** Content of individual coaching and psychoeducation group sessions.

Session	Individual behavioral activation coaching	Psychoeducation group
1	Discussion of stroke Introduction to treatment rationale Introduction to daily monitoring Important points about the structure of the treatment	Introduction to the workshop Introduction of group members Things that limit or enable what you want to do: inside you Things that limit or enable what you want to do: outside you Problem-solving Figuring out a home activity to work on
2	Daily monitoring: review assignment Treatment rationale: review Complete life areas, values, and activities inventory Complete activity selection and ranking	Planning what you want to do Taking apart the activity Problem-solving your home activity Taking apart the work, volunteer, or other service position Closing
3	Daily monitoring: review assignment Life areas, values, and activities inventory: review assignment Daily monitoring with activity planning and action planning	Figuring out important community activities Defining community participation Problem-solving meaningful work Identifying and problem-solving a community activity Requesting accommodations in the community Closing
4	Daily monitoring with activity planning and action planning: review assignment Daily monitoring with activity planning and action planning for the upcoming week Contracts	Feedback on the action plan developed with the coach earlier in the week Identifying meaningful work, volunteer, or service positions Requesting reasonable accommodations at work Decision-making Closing
5	Daily monitoring with activity planning and action planning: review assignment Contracts: review assignment Daily monitoring with activity planning and action planning for the upcoming week	Feedback on the action plan developed with the coach earlier in the week Making informed treatment decisions Communication skills Communicating with family and friends Working with your health care professionals and health care organization Closing
6	Daily monitoring with activity planning and action planning: review assignment Daily monitoring with activity planning and action planning for the upcoming week	Feedback on the action plan developed with the coach earlier in the week Dealing with depression Dealing with difficult emotions Positive thinking Closing
7	Daily monitoring with activity planning and action planning: review assignment Daily monitoring with activity planning and action planning for the upcoming week Life areas, values, and activities inventory: concept review and edit Activity selection and ranking: concept review and edit Contracts: concept review and edit Preparing for the end of treatment	Feedback on the action plan developed with the coach earlier in the week Relaxation techniques Looking back and planning for future Closing and wrap-up celebration

During the first 3 weeks, the coach worked with each participant to explore their valued life areas and activities and identify the treatment goals. A total of 25 predefined goals were programmed into iSMART. These goals target the application of self-management skills to improve participation in major life areas (ie, daily responsibilities, relationships, interests and recreation, education and career, and mind, body, and spirituality) derived from the behavioral activation treatment manual [31]. The coach entered up to 3 selected goals into the dashboard. Participants received the first SMS message at week 3, and for 10 consecutive weeks (ie, from week 3 to week 12), they received SMS messages every week. Multimedia Appendix 1 shows the screenshots of the iSMART dashboard. The dashboard can show the performance metrics of the study participants. It can also allow the coach to add, remove, or adjust goals and goal levels based on each survivor’s dynamic needs and preferences. The platform can automatically adjust self-management tips matched to the new goals sent to each survivor. In addition, the dashboard provides a secure chat feature that allows direct 2-way SMS text message communications between participants and the coach. All data collected via the apps, including SMS text messages sent or received through the secure chat function, were stored on Health Insurance Portability and Accountability Act–compliant servers behind the study institution’s firewall.

### Ethics Approval

The institutional review board of Washington University (202004137) and Northwestern University (STU00215743) approved this study.

### IM Approach

**Figure 3:**
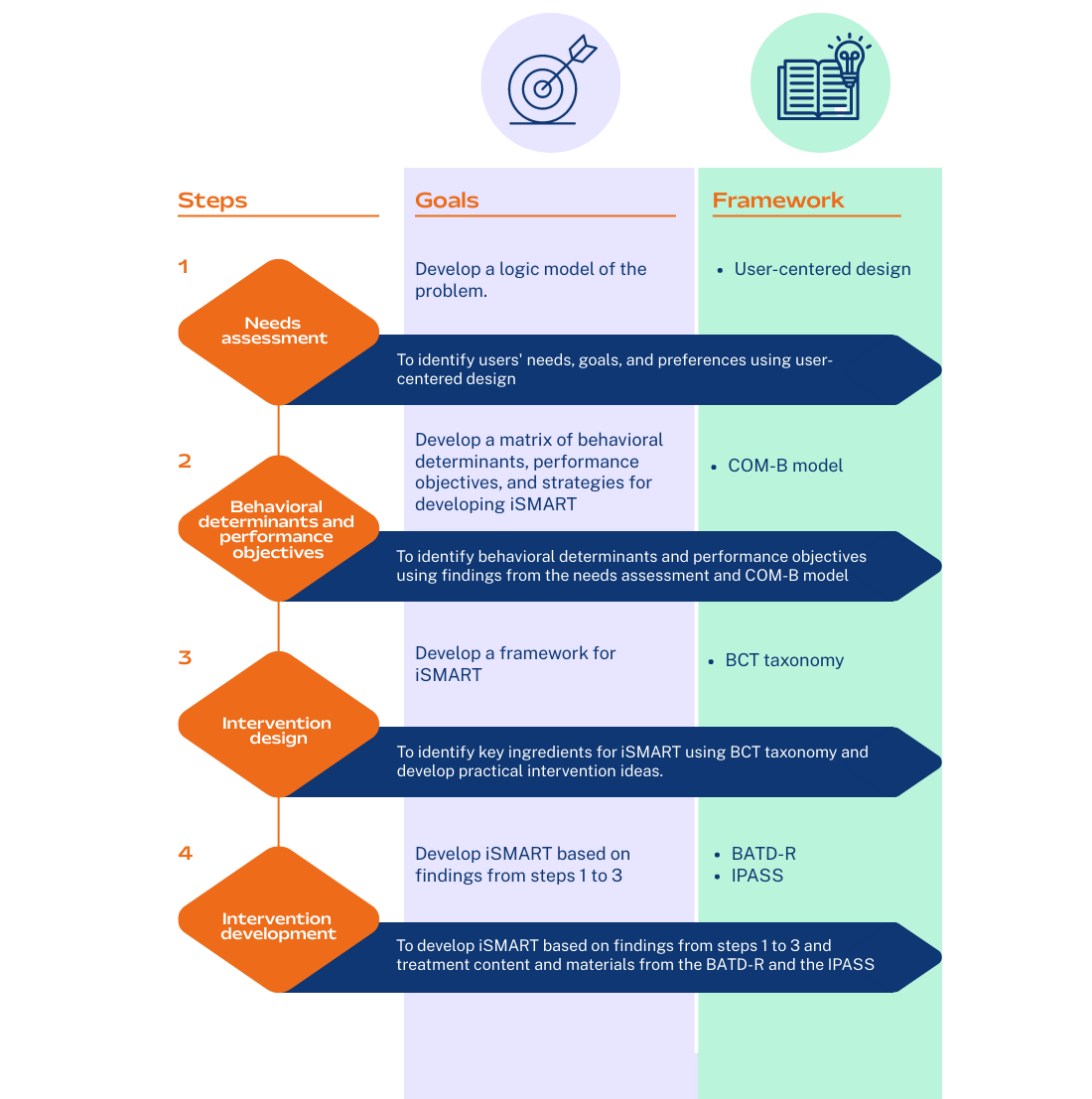
Intervention mapping steps. BATD-R: Brief Behavioral Activation Treatment for Depression-Revised; BCT: behavior change technique; COM-B: capability, opportunity, motivation, and behavior; IPASS: Improving Participation After Stroke Self-Management; iSMART: interactive Self-Management Augmented by Rehabilitation Technologies.

IM is a 6-step process often applied to guide the development, implementation, and evaluation of behavior change interventions [21,22]. It is a framework used to bridge the gap between theories and practice. Each step consists of several tasks, which, once completed, inform the next step. This rigorous framework ensures that the developed intervention can be implemented in real-world settings. The development process for iSMART was guided by the first four steps of IM, as illustrated in Figure 3, including (1) conducting a needs assessment, (2) specifying behavioral determinants and performance objectives, (3) applying theories and designing the intervention, and (4) developing and refining the intervention. Steps 5 (adoption and implementation plan) and 6 (evaluation plan) are being conducted in a separate clinical trial.

## Results

In this section, we outline the tasks used in each IM step and describe how these tasks were completed, followed by reporting key findings of each step.

### Step 1: Needs Assessment

#### Overview

Step 1 consisted of a detailed multimethod assessment of the needs of people after stroke to manage chronic conditions and participate in meaningful activities. This first IM step includes three tasks: (1) forming a planning group, (2) conducting a telephone survey, and (3) conducting a systematic review. The key findings of these tasks are provided in the following subsections.

#### Planning Group

The planning group (n=6) comprised stakeholders with expertise in stroke care, self-management, and technology development, including an occupational therapist in inpatient stroke rehabilitation, an occupational therapist in community rehabilitation, a director at a rehabilitation clinic, a director working at a technology company, a PhD-level nurse researcher with expertise in self-management, and a person living with chronic conditions after stroke. They had a wide range of experience in their content areas (mean 7.90, SD 5.44 years). They worked with the research team to adapt, test, and provide feedback on iSMART’s content, format, and perceived feasibility.

#### Telephone Survey

We conducted a telephone survey regarding mobile technology–supported health services with people after stroke (n=125). Participants were recruited from a stroke registry of patients admitted to a stroke center in the Midwestern United States. Our research assistants conducted telephone surveys with people after stroke about their use of mobile devices and preferences for technology-enabled services and formats for stroke rehabilitation. Survey questions were developed based on the Pew Research Center’s survey report regarding technology device ownership [35] and smartphone use [36], as well as a study of mobile technology services among persons with mental illness [37]. Multimedia Appendix 2 shows the survey questions. Please refer to our prior study [38] for participants’ eligibility and other details.

Of the 125 study participants, 79 (63.2%) were smartphone users (mean age 60.5, SD 13.0 years), and 46 (36.8%) were non–smartphone users (mean age 70.5, SD 10.9 years). Of the 79 smartphone users, 39 (49%) were men, and 44 (56%) were White; whereas, of the 46 non–smartphone users, 24 (52%) were men, and 33 (72%) were White. The top 5 desired services rated by smartphone users were appointment or activity scheduling, symptom management, stroke prevention, cognitive enhancement training, and access to stroke care resources. Non–smartphone users also rated these 5 services as their most desired services, plus socialization, as their most desire services. Smartphone users also reported that making video or voice calls, SMS text messaging, and surfing the internet were the top 3 most common functions associated with their mobile device use over the last 12 months. On the basis of these findings, we decided to focus the iSMART content on helping participants to identify and schedule their valued activities into their daily routine; remind them about, and monitor the progression of, goal completion; monitor and manage symptoms; and provide resources and knowledge associated with self-management and secondary stroke prevention. For the iSMART’s delivery format, we used videoconferencing to run the psychoeducation and coaching sessions and SMS text messaging (with a clinician-facing dashboard to customize SMS text messages to participants) to support self-management behaviors.

#### Literature Review and Meta-Analysis

**Figure 4:**
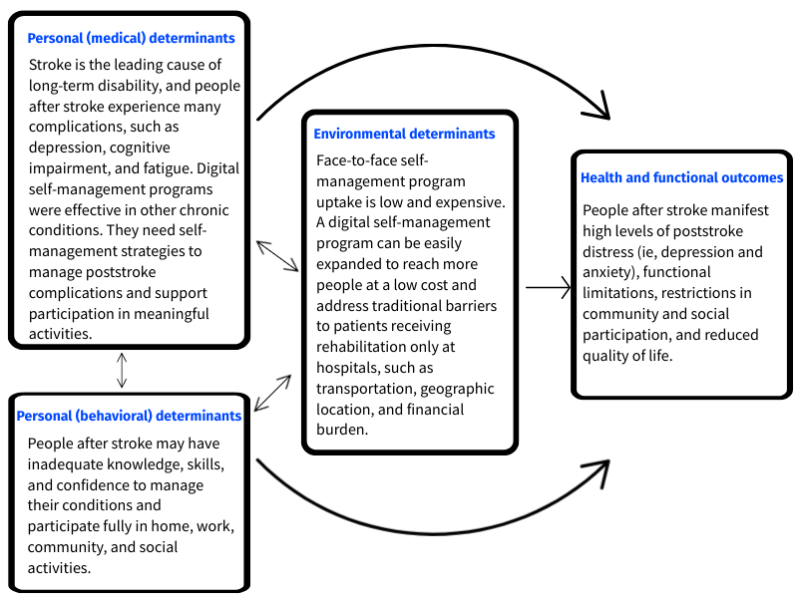
Logic model of the problem.

We conducted a literature review to identify personal (medical and behavioral) and environmental factors associated with the inability to manage chronic conditions and symptoms leading to restricted participation in meaningful activities. We summarized and produced a logic model (Figure 4) to link personal (medical and behavioral) determinants and environmental determinants, leading to the target health and participation problems.

We also conducted a meta-analysis study of digital self-management interventions in people with neurological disorders. The review aimed to identify the theory, outcomes, and optimal mode of intervention delivery for developing iSMART. A prior manuscript describes our detailed search strategy, study selection, data extraction, quality appraisal, and statistical analyses [19]. We found that interventions based on cognitive behavioral theory were effective in reducing depression, anxiety, and fatigue and enhancing self-efficacy. By contrast, interventions based on social cognitive theory were effective in reducing depression only. In addition, digital self-management interventions that incorporated live health coaching or support from a health professional were found to be more effective than fully digital self-guided interventions. Thus, we incorporated live health coaching into the digital intervention framework, facilitated through scheduled videoconferences and both scheduled and ad hoc bidirectional SMS text messages.

### Step 2: Behavioral Determinants and Performance Objectives

Informed by the findings from the needs assessment, we used the capability, opportunity, motivation, and behavior model [39] to guide the identification of key behavioral determinants of self-management. We then derived performance objectives that mapped to each behavioral determinant. Performance objectives are actions taken by people after stroke to achieve behavioral determinants.

The study team, which consisted of experts in digital health intervention development, adaptation, and testing, identified seven behavioral determinants most likely to affect the treatment goal and outcomes: (1) knowledge, (2) behavioral regulation, (3) skills, (4) self-efficacy, (5) motivation, (6) negative and positive affect, and (7) social support and environmental support. We further derived performance objectives mapped to each behavioral determinant (Textbox 1).

**Behavioral determinants and performance objectives.**

**Knowledge**

- Learn about stroke consequences, benefits of the intervention, factors, and resources available to support self-management behaviors and activity participation

**Behavioral regulation**

- Monitor progress in increasing self-management behaviors and meaningful activity participation

**Skills**

- Learn self-management skills and strategies

**Self-efficacy**

- Increase confidence to use self-management strategies effectively

**Motivation**

- Increase engagement in planned goals and activities and engage with the digital program

**Negative and positive affect**

- Cope with challenges associated with self-management behaviors and engagement in planned activities

**Social support and environmental support**

- Provide resources to peers and increase access to resources

### Step 3: Intervention Design

We developed a framework to identify target behaviors and outcomes, considering the MoAs most likely to affect the selected behavioral determinants. MoAs are constructs that can be individual characteristics (eg, knowledge and attitudes) or contextual characteristics (eg, social support and environmental resources) to mediate intervention effects [40]. Next, we used the linkage table published by Carey et al [40] to match the behavior change techniques (BCTs) to each of the MoAs. Subsequently, the planning group members developed by consensus a set of practical empirically supported approaches to address the selected BCTs.

**Figure 5:**
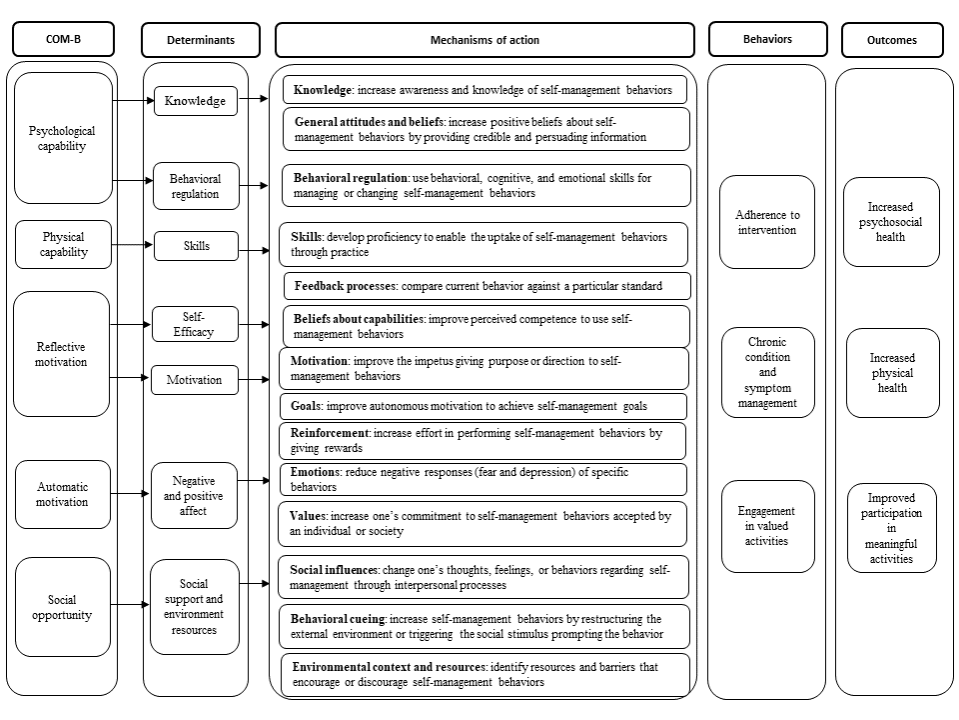
The theoretical framework of behavior changes used to inform the design, additional content, and functionality of the interactive Self-Management Augmented by Rehabilitation Technologies (iSMART) intervention. COM-B: capability, opportunity, motivation, and behavior.

Figure 5 outlines the theoretical framework used to inform the design, additional content, and functionality of iSMART. We identified 14 MoAs affecting behaviors (ie, adherence to the intervention, chronic condition and symptom management, and engagement in valued activities), ultimately leading to improved outcomes (ie, physical health, psychosocial health, and activity participation) for people with disabilities. Multimedia Appendix 3 describes the MoAs, BCTs, and practical application ideas used for iSMART development.

### Step 4: Intervention Development

#### Overview

We developed all iSMART components based on the findings from steps 1 to 3. For the psychoeducation component, we incorporated the practical application ideas to adapt the original IPASS content into iSMART. For the coaching component, we incorporated the Brief Behavioral Activation Treatment for Depression-Revised manual [31] into iSMART. For the SMS text messaging component, we adapted an SMS platform used in previous studies [32-34], including a clinician-facing dashboard and SMS text messaging libraries. Although the iSMART SMS text messaging followed the same weekly messaging schedule as the parent platform, we developed new SMS text message content customized to improve skills in managing chronic conditions and support participation in daily life and community activities for people after stroke. Next, the planning group members reviewed all materials, including the iSMART manual, treatment forms, and SMS text messages. A health literacy specialist also reviewed these materials to improve health literacy elements and readability. We revised the treatment materials and liaised with a digital health start-up to refine the digital platform. Afterward, each of the planning group members trialed the refined program for 2 to 3 weeks and completed three 4-item implementation outcome measures [41]—the Acceptability of Intervention Measure (AIM), the Intervention Appropriateness Measure (IAM), and the Feasibility of Intervention Measure (FIM)—to assess the preimplementation feasibility of iSMART. Items were rated on a 5-point scale, ranging from 1=completely disagree to 5=completely agree. Benchmarks for high acceptability, appropriateness, and feasibility of the study intervention are mean scores of 4 out of 5 on the AIM, IAM, and FIM. These measures had strong structural validity with Cronbach α values of .85 for the AIM, .91 for the IAM, and .89 for the FIM as well as strong test-retest reliability with Cronbach α values of .83 for the AIM, .87 for the IAM, and .88 for the FIM [41]. The planning group members also provided written feedback for further improvement.

We have reported the intervention architecture and theoretical foundations of iSMART in the *Methods* section. We focus on reporting major changes to iSMART in the *Results* section.

#### Major Changes to iSMART

The planning group members suggested minor but important changes to the order in which content is delivered to improve iSMART. First, they suggested reorganizing the psychoeducation content. Specifically, they recommended moving the problem-solving module to the first session so that participants could learn these foundational skills before attempting more challenging skills, such as requesting accommodations. They also suggested that the *dealing with depression* content should be delivered before the *positive thinking* *and dealing with difficult emotions* content. It was hypothesized that this reorganized learning sequence would avoid people experiencing depressive symptoms to find strategies learned via *positive thinking and dealing with difficult emotions* content less effective. They also recommended that home-based content be delivered before community-based content, allowing participants to practice their skills in a more familiar setting. Second, the planning group members advised dividing the 15-minute break into one 5-minute break and one 10-minute break during group sessions to optimize participants’ attention and engagement. Third, to reduce participant burden, the planning group members recommended that if the coach chose to implement iSMART in a one-on-one format, the coach could select relevant self-management psychoeducation modules based on the participant’s needs instead of teaching all 5 modules.

Finally, the planning group members suggested adjusting the time commitment for completing the different treatment forms or homework. Instead of asking participants to report hourly activities 7 days a week, they proposed that the coach and participant select 2 days of the week for reporting activities that best represent their regular rescheduling within the first 3 coaching sessions. In addition, the planning group members voiced concern that participant comfort and familiarity with mobile health technology and SMS text messaging would potentially limit engagement and affect the intervention’s overall effect. Thus, we developed simplified educational content to support the use of common digital tools (eg, videoconference tool) on mobile devices.

#### Preimplementation Feasibility of iSMART

The planning group members scored the acceptability, appropriateness, and feasibility (on a scale of 1 to 5, with 5 being most favorable) of the iSMART program as high, with mean scores of 4.63 (SD 0.38), 4.63 (SD 0.38), and 4.58 (SD 0.34), respectively. These findings suggest that our iSMART is an acceptable, appropriate, and feasible program.

## Discussion

### Principal Findings

In this study, we present a use case of the application of IM and BCTs to adapt and develop a digital intervention to improve the self-management of chronic conditions and daily activity participation in people after stroke. Although digital behavioral interventions are becoming increasingly popular, more research is needed to guide the process of translating evidence, theories, existing interventions, and user feedback for use in developing or adapting digital behavioral interventions. This study can serve as a design blueprint for researchers aiming to digitize self-management or other behavioral programs to improve intervention access, engagement, and effectiveness. This study has applied multiple empirically supported theories and stakeholder input to inform intervention development (using the IM framework to organize intervention inputs), produce an overarching logic model, and identify MoAs and BCTs to guide intervention development.

### Prior Works and Study Implications

The technology-supported delivery of behavioral interventions holds promise for improving the precision of behavioral interventions by allowing the intervention to be tailored to the user and adapted over the course of the intervention as the user makes progress [42]. The iSMART platform uses an innovative architecture that facilitates personalized interaction and accessible resources provided to the user on demand.

As in the case of other chronic conditions, people after stroke often face challenges with limited access to needed health services [43]. iSMART uses SMS text messaging and videoconferencing, which can provide an alternative delivery solution to those who would not otherwise have access to these essential services [14]. The iSMART intervention can be expanded to reach many mobile phone users at a low cost and address clinical barriers to access. As of 2021, about 97% of American adults own a mobile phone, and 85% own a smartphone [44]. In addition, 73% of mobile phone owners use SMS text messaging on their mobile phones, and these SMS text messaging users send or receive an average of 41.5 messages per day [45]. Those with lower income (ie, <US $30,000 per year) and education (ie, ≤high school) mainly rely on their mobile phones for web-based access [46]. Digital interventions can overcome traditional barriers to patients receiving rehabilitation only at hospitals, such as inconvenience, geographic isolation, and financial burden [47].

### Limitations and Future Directions

Because of the funding constraint, the planning group is small, and only 1 person with stroke has been included. Recognizing that patient and public involvement is essential to strengthen and improve the quality of the tool and make it more relevant [48], future research should involve more people after stroke and their caregivers in the development process.

The planning group makeup is such that it does not represent individuals from populations that may be at higher risk for chronic health conditions associated with stroke risk, including populations with low literacy; those who are socioeconomically disadvantaged and may have limited access to, or experience using, technology; and underrepresented communities, such as racial, ethnic, and gender minority groups. Adaptations to the iSMART intervention may not fully cover the unique needs of these vulnerable populations. Future steps include engaging a more heterogeneous population of people after stroke or persons with other disabilities in user-centered design activities that may increase the adoption and sustainable implementation of iSMART to a broader population of end users. The use of IM for intervention development is a resource-intensive process, consuming substantial efforts and resources. Nonetheless, we found the IM to be a valuable framework for guiding interventions for the target population.

### Conclusions

This paper demonstrates the use of IM and BCTs to support the adaptation and development of an intervention designed to promote poststroke self-management skills to improve the management of chronic conditions and promote daily life participation. The rigorous process results in higher transparency in understanding treatment mechanisms and allows replications for designing other complex interventions. This paper can be a valuable blueprint for developing digital interventions for self-management in different conditions.

## Appendix

Multimedia Appendix 1 Screenshots of the interactive Self-Management Augmented by Rehabilitation Technologies (iSMART) dashboard.

Multimedia Appendix 2 Survey questions.

Multimedia Appendix 3 Mechanisms of action linked to behavior change techniques and the design of practical applications.

## Abbreviations

AIM: Acceptability of Intervention Measure
BCT: behavior change technique
FIM: Feasibility of Intervention Measure
IAM: Intervention Appropriateness Measure
IM: intervention mapping
IPASS: Improving Participation After Stroke Self-Management
iSMART: interactive Self-Management Augmented by Rehabilitation Technologies
MoA: mechanism of action

## Data Availability

The data generated and analyzed during this study cannot be sufficiently deidentified and, therefore, cannot be made publicly available owing to ethical considerations. Deidentified data can be made available, for further research, by the corresponding author on reasonable request.
