# Supplementary material for: Using Intervention Mapping and Behavior Change Techniques to Develop a Digital Intervention for Self-Management in Stroke: Development Study
Source: JMIR Hum Factors. 2023 Jul 24;10:e45099. doi: 10.2196/45099 (PMC10407772; doi:10.2196/45099)

Multimedia Appendix 1 - Screenshots of the interactive Self-Management Augmented by Rehabilitation Technologies (iSMART) dashboard.

(1) Start-up page:

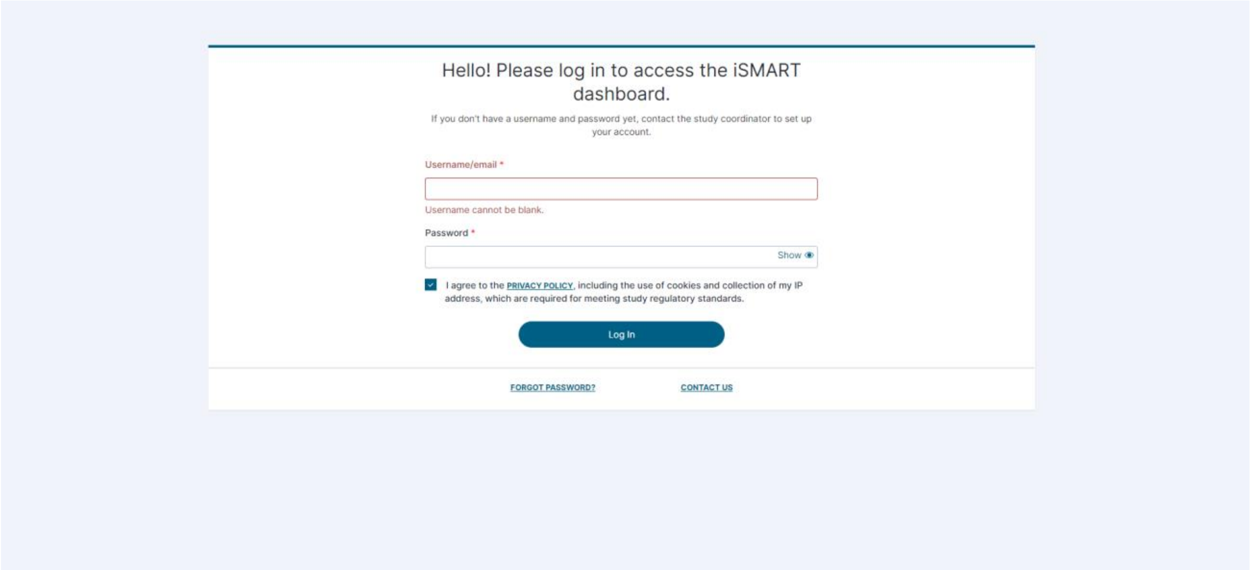

(2) Study overview with performance metrics for all participants

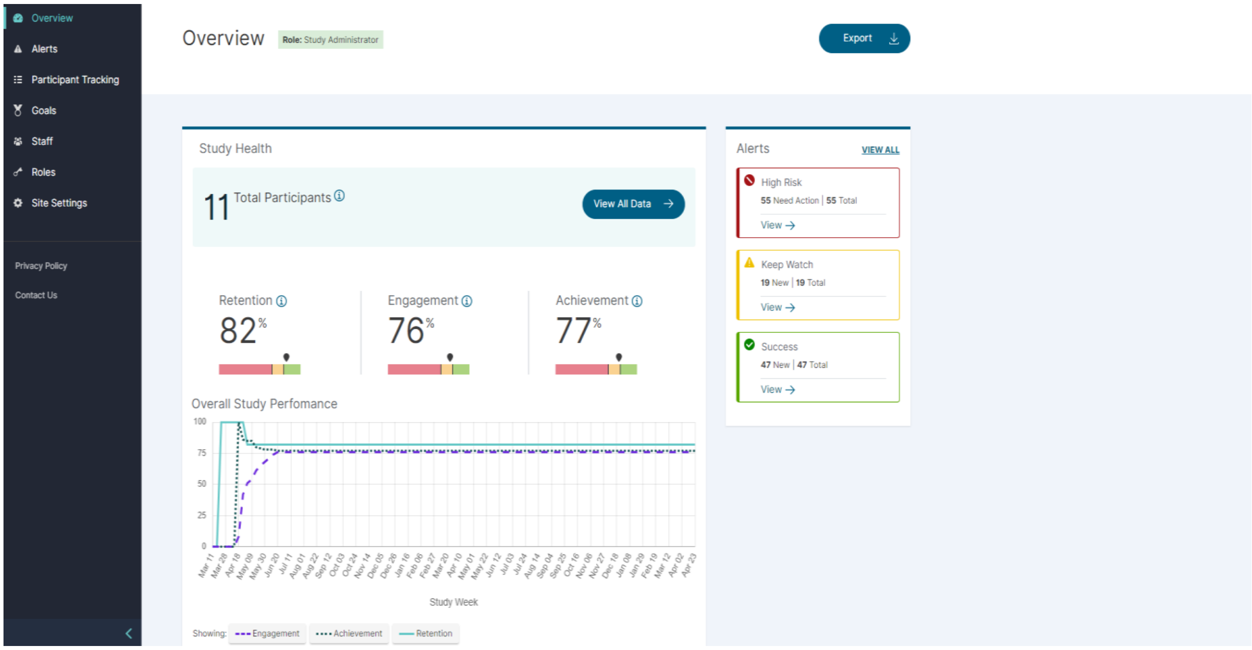

(3) Study overview with performance metrics by goals for all participants

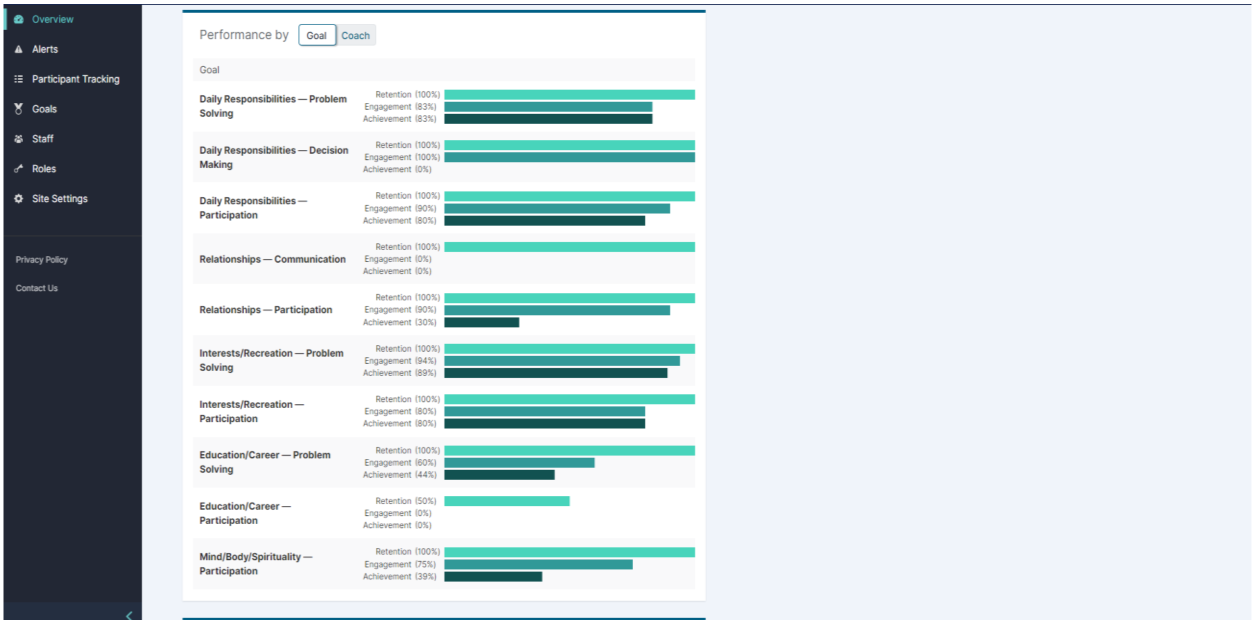

(4) Participant tracking for all participants

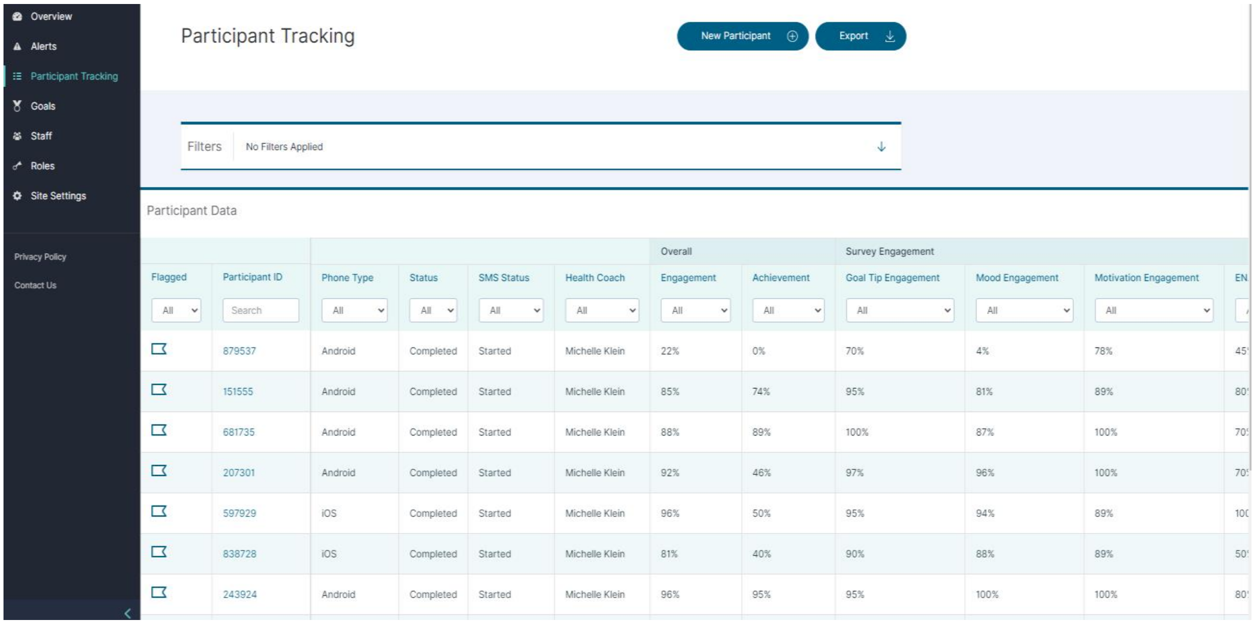

(5) Participant tracking with overall performance metrics and mood for a selected participant

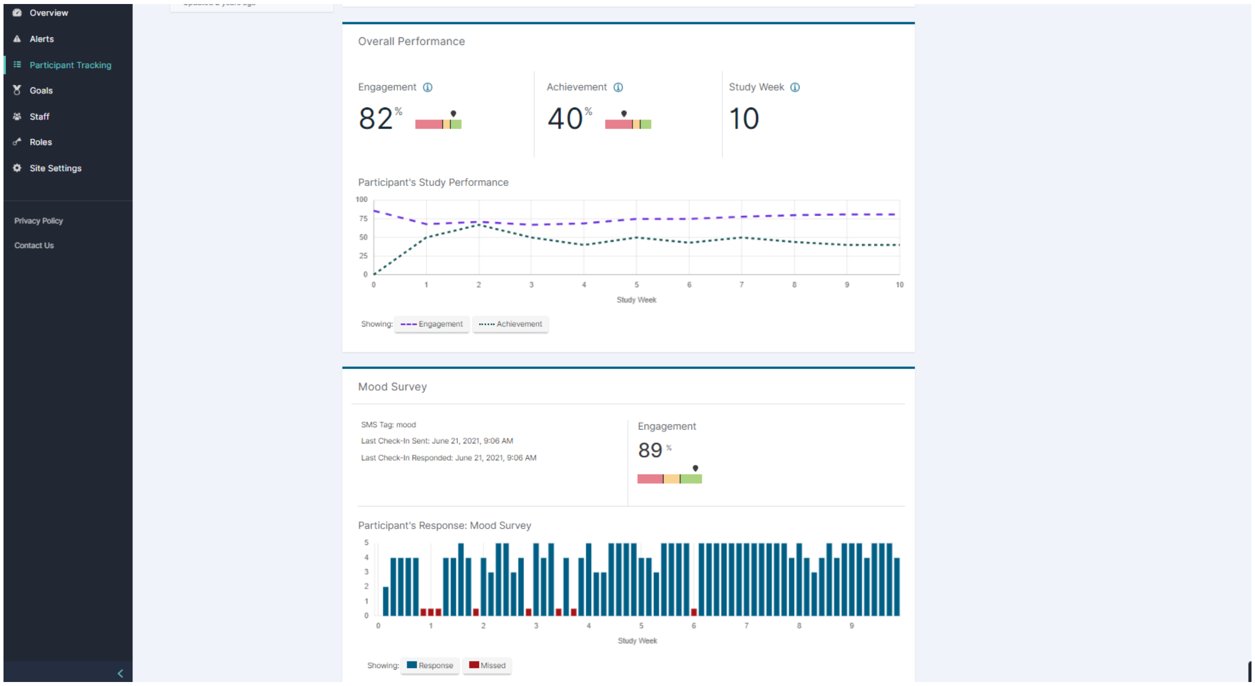

(6) Participant tracking with performance metrics by goals for a selected participant

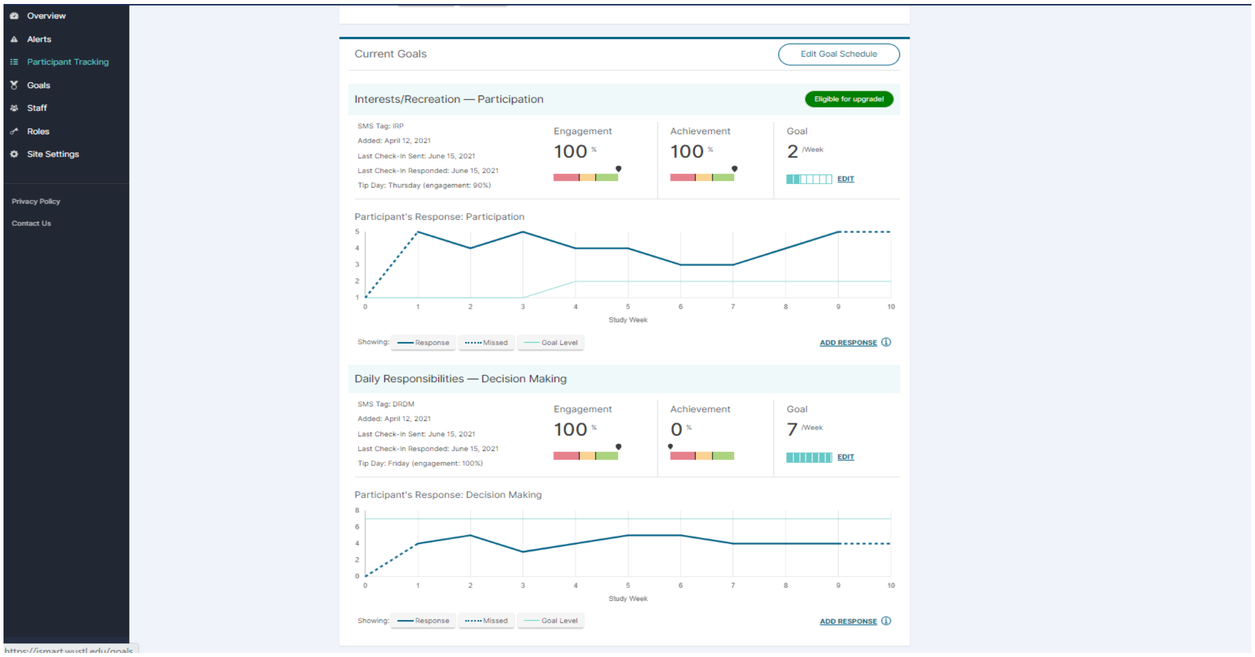

(7) Alert page showing participants who need the coach's attention

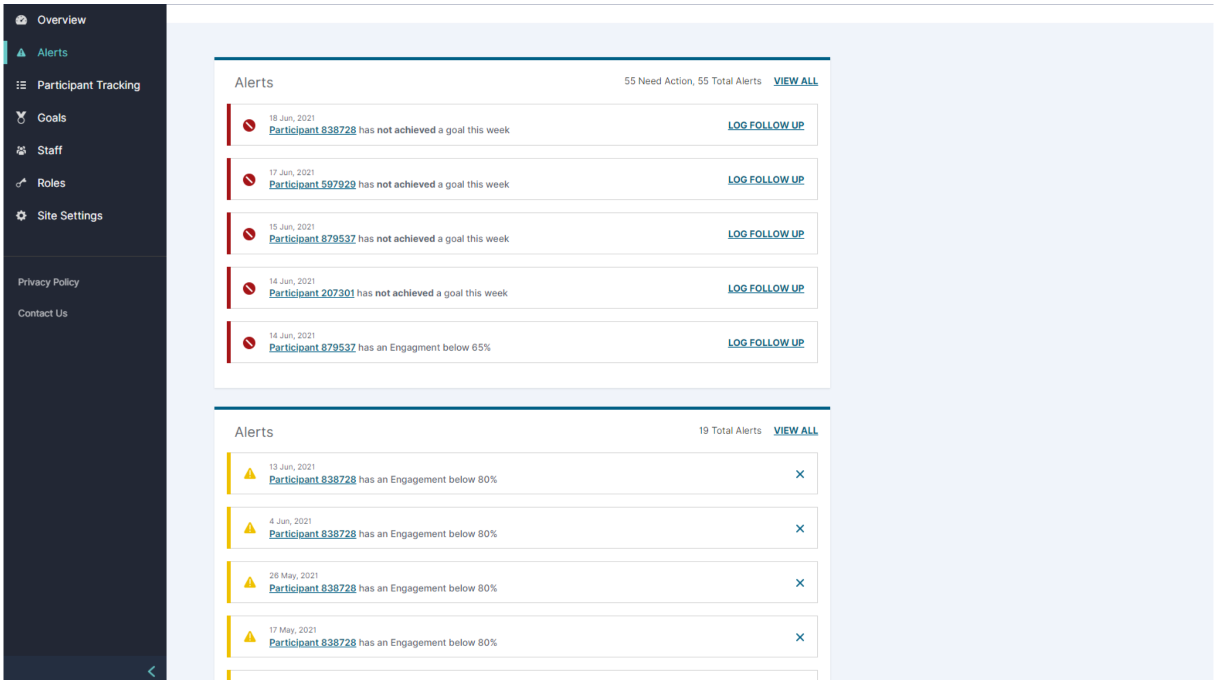

(8) Chat function for participant-coach communications

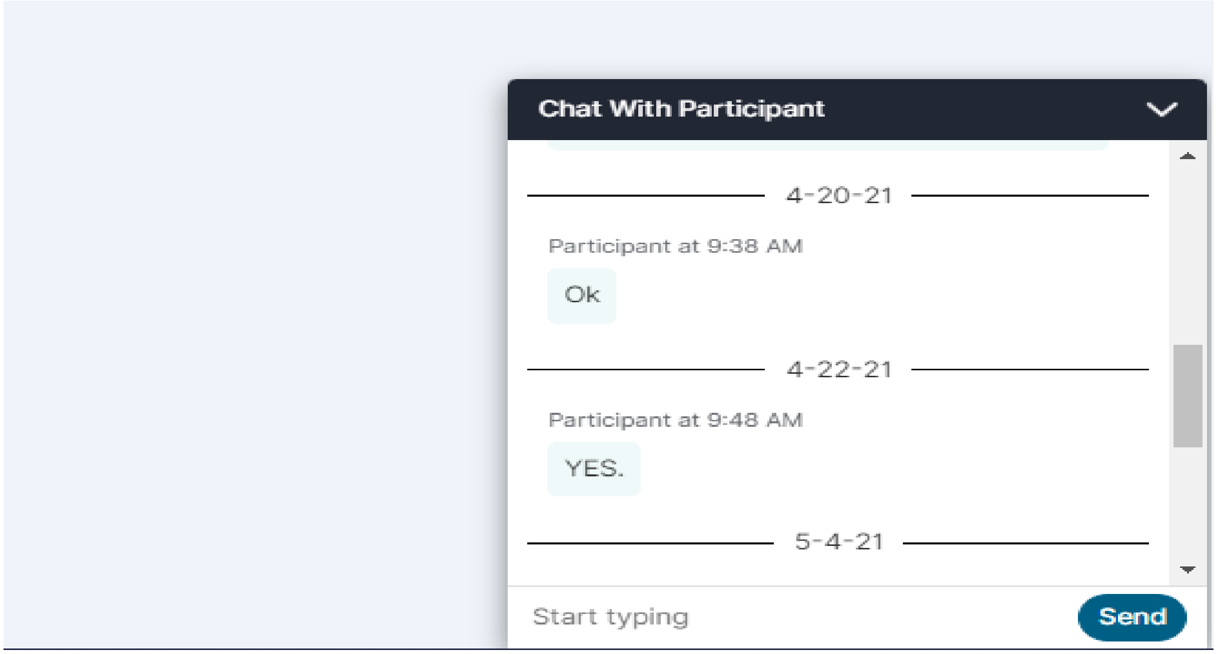

Supplement: Multimedia Appendix 1 [file humanfactors_v10i1e45099_app1.pdf]
