# Supplementary material for: Using Intervention Mapping and Behavior Change Techniques to Develop a Digital Intervention for Self-Management in Stroke: Development Study
Source: JMIR Hum Factors. 2023 Jul 24;10:e45099. doi: 10.2196/45099 (PMC10407772; doi:10.2196/45099)
Supplement: Multimedia Appendix 2 [file humanfactors_v10i1e45099_app2.pdf]

Multimedia Appendix 2 – Survey questions

[For all people]

Do you have a cell phone or not? (Yes/No/Don't know/Refused)

Some cell phones are called "smartphones" because of certain features they have. Is your cell phone a smartphone such as an iPhone, Android, Blackberry, or Windows phone, or are you not sure?

- Yes, it is a smartphone
- No, it is not a smartphone
- Don't know
- Refused

Which of the following best describes the type of cell phone you have? Is it an iPhone, Blackberry, Android, Windows Phone, or something else? (If you have multiple phones, select the one you use most often)

- Android
- iPhone
- Windows
- Blackberry
- Basic cell phone
- Something else
- Don't know
- Refused

Please tell me if you have each of the following items or not. Do you have… (Yes/No/Don't know/Refused)

- Smartphone
- Desktop or Laptop computer
- Tablet computer, e.g., iPad, Samsung Galaxy Tab, Google Nexus, or Kindle Fire
- Game console, e.g., Xbox or Play Station
- Portable gaming device, e.g., PSP
- MP3 player or iPod
- E-book reader, e.g., Nook or Kindle e-reader

Which of the following is your primary device (i.e., the one you use the most)?

- Cell phone (Non-smartphone)

- Smartphone
- Desktop or Laptop computer
- Tablet computer
- Game console
- Portable gaming device
- MP3 player
- E-book reader
- Other_______

How often do you use this primary device?

- Daily
- Weekly
- Monthly
- Less than once a month

Which of the following have you done in the last year by using your mobile device? (Check all that apply)

- Voice/video calls
- Text messaging
- Internet browsing
- Look up information about a job (e.g., job search, access career opportunities)
- Submit a job application
- Access government services or information
- Take a class or watch educational content
- Get information about a health condition
- Look up real estate listings or information about a place to live
- Do online banking (for example, pay a bill or transfer money)
- Follow breaking news
- Share pictures, videos, or commentary with others about events happening in your community
- Make a monetary donation to a political or charitable cause
- Learn about events and activities in your community
- Turn-to-turn driving navigation
- Get public transit information
- Reserve a taxi or car service

What is your primary device ownership?

- Individual ownership
- Shared
- Rented
- Borrowed
- Others: please specify _________________

What is your current payment plan?

- Month-to-month
- Government plan (e.g., Federal Lifeline Program that helps qualified low-income individuals pay for mobile phone service)
- Contract plan
- Prepaid card

Do you have broadband (Internet) service at home?

- Yes
- No
- No answer

Do you have mobile broadband (Internet) service (e.g., 4G/3G)?

- Yes
- No
- No answer

Which of the following statements comes closest to describing how you use your cell phone to access online services and information, even if neither is exactly right?

- Other than my cell phone, I have a limited number of ways to get online
- I have a number of other options for getting online in addition to my cell phone
- No answer

What other options are available to access online services and information besides your cell phone? Access (e.g., WIFI) at ……. (Check all that apply)

- Friend or neighbor's home
- Relative's home
- Work Office
- Public transit
- Restaurant or Café

- Library or other public buildings
- Others: Please specify ___________________

Which of the following statements most closely matches how you feel about your primary device, even if neither is right?

- Helpful
- Annoying
- No answer

Which of the following statements most closely matches how you feel about your primary device, even if neither one is exactly right?

- Worth the cost
- Financial burden
- No answer

Which of the following statements most closely matches how you feel about your primary device, even if neither one is exactly right?

- Connecting
- Distracting
- No answer

Which of the following statements most closely matches how you feel about your primary device, even if neither one is exactly right?

- Freedom
- Leash
- No answer

Which of the following statements most closely matches how you feel about your primary device, even if neither one is exactly right?

- Couldn't live without
- Not always needed
- No answer

[For smartphone users or tablet users only]

We will ask some questions about your Smartphone/Tablet use

Which one of the following features do you use most frequently? Please choose one only

- Text messaging
- Voice/video calls
- Email
- Internet use
- Social networking
- Take pictures/video
- Follow news
- Games
- Listening to music or podcasts
- Watching video
- Using maps and navigation
- Paying bills
- Others

Which one of the following features do you use second most frequently? Please choose one only

- Text messaging
- Voice/video calls
- Email
- Internet use
- Social networking
- Take pictures/video
- Follow news
- Games
- Listening to music or podcasts
- Watching video
- Using maps and navigation
- Paying bills
- Other

Which of the following emotions have you felt the most due to having a smartphone? Please choose one only

- Productive

- Happy
- Grateful
- Inspire positive feelings
- Distracted
- Angry
- Frustrated

Which of the following emotions have you felt the second most due to having a smartphone? Please choose one only

- Productive
- Happy
- Grateful
- Inspire positive feelings
- Distracted
- Angry
- Frustrated

In which of the following locations have you used your device the most? Please choose one only

- At home
- In a car or public transit
- At work
- Waiting in line
- At a community place
- Walking from place to place
- Exercising
- Other

In which of the following locations have you used your device the second most? Please choose one only

- At home
- In a car or public transit
- At work
- Waiting in line
- At a community place
- Walking from place to place
- Exercising
- Other

[For those who do not have a mobile device, ask:]

What is the primary reason for not possessing a mobile device?

- Financial constraints
- Lack of interest
- Lack of necessity
- Do not know how to use a mobile device
- Unable to use a mobile device due to the motor disability
- Unable to use a mobile device due to the cognitive limitation
- Unable to use a mobile device due to the visual problem
- Unable to use a mobile device due to the hearing problem
- Others: please specify _________________

[For all people]

Below we will ask a few questions about your interest in using mobile health services. Are you interested in trying mobile health services (if we provide the training)? (yes/no)

What services do you want to be provided through mobile technology? (Can choose more than 1)

- Symptoms/ Chronic disease management
- Medication adherence
- Medical appointment schedule and reminders
- Socialization
- Prevention of stroke recurrence
- Cognitive Enhancement
- Mood Regulation
- Resources and information for social/community services related to stroke care
- Resources and information for social/community services for supported employment, housing, & transportation
- Lifestyle and health promotion (e.g., healthy diet, exercise)

What medium of service delivery do you prefer to receive? (Can choose more than 1)

- Apps
- Calls
- Text messages
- Email
- Internet
- Social media

Are you interested in joining a focus group to provide suggestions while we develop and test the technology-based devices and services? (yes/no)

Are you interested in participating in research while we deliver the technology-based health services study? (yes/no)

Would you prefer to use your mobile device to participate in the research study about mobile health services?

- Yes, I am happy to join the study using my device
- No, I will decline the study even if the money compensation is provided
- No, I prefer to use the university-borrowed device (even though I need to carry my phone and the university-borrowed phone during the study period)

If we design a survey and ask questions via your mobile device, each survey will last 5-10 minutes (for 25-30 questions); how often do you feel acceptable and willing to participate?

- 10 times/day x 5 days
- 8 times/day x 7 days
- 6 times/day x 7 days
- 5 times/ day x 7 days
- 4 times/day x 10 days
- 3 times/day x 10 days

How much do you feel is appropriate for the schedule you chose as your participation compensation?

$ ___________

Will you join the study if we ask you to wear a motion sensor, e.g., a pedometer, like wearing a watch on your wrist for tracking the level of physical activity? (yes/no)

Will you join the study if we ask you to enable your device's GPS for tracking your locations? (yes/no)

How do you describe your current residence?

- Urban (population over 1,000,000)
- Suburban (population between 10,000 and 1,000,000)
- Rural (population under 10,000)
- Don't know
- Refused

What is your annual household income?

- <$30K
- $30K-$49,999
- $50K-$74,999
- $75K+
- Don't know
- Refused
