# Supplementary material for: Using Intervention Mapping and Behavior Change Techniques to Develop a Digital Intervention for Self-Management in Stroke: Development Study
Source: JMIR Hum Factors. 2023 Jul 24;10:e45099. doi: 10.2196/45099 (PMC10407772; doi:10.2196/45099)
Supplement: Multimedia Appendix 3 [file humanfactors_v10i1e45099_app3.pdf]

Multimedia Appendix 3. Mechanisms of action (MoAs) linked to behavior change techniques (BCTs) and the design of practical applications.

MoAs	BCTs[a]	Practical application ideas	Description of implementation strategies
Knowledge	• 5.1: information about health consequences<br>• 5.3: information about social and environmental consequences<br>• 4.1: instruction on how to perform the behavior<br>• 2.1: feedback on behavior	• Self-guided educational materials<br>• Facilitated group discussion<br>• Homework assignments<br>• SMS text messages for reminders and support[b]	• We provided didactic education and materials about stroke, poststroke consequences, and meaningful activity participation, including the benefits of active engagement in valued activities and self-management<br>• We sent daily text messages related to self-management tips[b]
General attitudes and beliefs	• 9.1: credible source	• Health coach training and education<br>• Patient onboarding and education<br>• Patient feedback on intervention components via SMS text message[b]	• We developed a treatment manual and materials based on credible sources such as the American Stroke Association and evidence-based information from peer-reviewed articles<br>• We sent SMS text messages asking participants to rate whether the messages for self-management tips or motivation are helpful[b]
Behavioral regulation	• 2.3: self-monitoring of behaviors<br>• 1.4: action planning<br>• 8.4: habit reversal<br>• 8.2: behavior substitution	• Patient-facing education and treatment materials<br>• Life areas, values, and activities form<br>• Activity selection and ranking form<br>• Action plan form	• We created treatment content focusing on specific skills (problem-solving, decision-making, emotion and symptom management, communication, and accommodations)

	• 1.1: goal setting (behavior)<br>• 1.6: discrepancy between current behavior and goal<br>• 2.4: self-monitoring of outcomes of behaviors<br>• 8.3: habit formation	• Reflection form<br>• Goal-specific SMS text messages[b]	• Forms were developed to aid in prioritizing activities and setting personal goals<br>• We identified strategies or resources needed to achieve target goals<br>• We identified problems while performing activities<br>• We sent semiautomated, goal-specific reminders and check-in messages via SMS text messages to track progress and identify challenges[b]
Skills	• 8.1: behavior practice/rehearsal<br>• 6.1: demonstration of the behavior<br>• 4.1: instruction on how to perform the behavior<br>• 1.2: problem-solving<br>• 8.6: generalization of the target behavior	• Educational materials for group-based treatment delivery<br>• Instructions/tools for skills demonstration by the leader (video/sound scripts)<br>• Action plan form<br>• Reflection form<br>• SMS text messages[b]	• We developed group-session scripts for behavioral demonstration<br>• We also developed videos and scripts for specific skills (eg, relaxation)<br>• We provided action plan and reflection forms to identify strategies or resources needed to achieve goals and identify problem areas<br>• We sent SMS text messages about self-management skills[b]
Feedback processes	• 9.2: pros and cons<br>• 2.7: feedback on outcomes of behaviors	• Feedback from the group leader and peers<br>• SMS text messages[b]	• We developed warm-up activities to help peers to build rapport, facilitate sharing, and provide feedback, and we encouraged health coaches to complete the reflection form to give participants feedback

			• We sent SMS text messages to inform participants when they missed or met the goals[b]
Beliefs about capabilities	• 8.7: graded tasks<br>• 15.1: verbal persuasion about capability<br>• 15.3: focus on past success<br>• 6.1: demonstration of the behavior<br>• 1.2: problem-solving<br>• 8.1: behavioral practice/rehearsal<br>• 11.2: reduce negative emotions	• Educational and treatment materials<br>• Group discussion<br>• Structured practice by participants<br>• Feedback on perceived capabilities from the group leader and peers	• We developed treatment content for teaching problem-solving, decision-making, emotion and symptom management, communication, and accommodations<br>• Participants practiced the skills learned during the group discussion<br>• The coach and peers provided feedback
Motivation	• 15.2: mental rehearsal of the successful performance<br>• 9.2: pros and cons<br>• 13.5: identity associated with changed behaviors<br>• 10.7: self-incentive	• Action plan form<br>• Reflection form<br>• Feedback from the leader and peers<br>• SMS text messages[b]	• The coach and peers encouraged other participants to continue their action plans and behavior changes<br>• We also provided the action plan form and the reflection form for the coach to identify strategies to motivate participants for self-management and provide feedback for problems experienced by participants<br>• We sent weekly motivational SMS text messages to encourage self-management practice[b]
Goals	• 1.6: discrepancy between current behavior and goal	• Action plan form<br>• Reflection form<br>• Feedback from the leader and peers	• We provided the action plan and the reflection form to review progress with the participants and provide feedback

	• 1.3: goal setting (outcome)<br>• 1.7: review outcome goals	• SMS text messages[b]	• The coach and peers provided feedback during the group discussion<br>• We sent SMS text messages to participants when they missed or met the goals[b]
Reinforcement	• 10.3: nonspecific reward<br>• 10.4: social reward<br>• 7.8: associate learning	• Action plan form<br>• Reflection form<br>• Feedback from the leader and peers<br>• SMS text messages[b]	• The coach and peers encouraged other participants to continue their action plans and behavior changes<br>• The coach reviewed the action plan and the reflection form with participants to appraise their achievements<br>• We sent motivational SMS text messages when participants met or exceeded goals[b]
Emotions	• 5.5: anticipated regret<br>• 5.6: information about emotional consequences	• Educational materials<br>• Depression protocol<br>• Community mental health resources handout<br>• SMS text messages[b]	• We developed treatment content for teaching skills to cope with distress and manage emotions, and we also developed videos and scripts for specific skills (eg, relaxation) to cope with stress<br>• We developed a protocol for responding to participant report of depressive symptoms<br>• We created a community resources page to identify available mental health services in local areas<br>• We sent daily mood check-in SMS text messages to assess daily mood levels[b]

Values	• 13.5: identity associated with changed behavior<br>• 15.2: mental rehearsal of the successful performance<br>• 1.9: commitment	• One-on-one coaching<br>• Group discussion<br>• Activity list<br>• Action plan worksheet	• The coach worked with participants individually and in a group setting to identify their values and activities associated with these values<br>• We developed the activity list to facilitate participants to identify valued activities that they can engage in<br>• We used the action plan form for participants to build solid plans to meet the goal
Social influences	• 3.1: social support (unspecified)<br>• 12.2: restructuring the social environment<br>• 3.2: social support (practical)<br>• 2.1: monitoring of behavior by others without feedback<br>• 6.2: social comparison	• Group discussion<br>• Feedback and support from the group leader and peers	• We provided a secure environment for participants to share their thoughts<br>• Participants shared experiences of their journey<br>• The coach and peers encouraged other participants to continue with their action plans and behavior change
Behavioral cueing	• 8.3: habit formation<br>• 7.1: prompts/cues<br>• 12.1: restructuring the physical environment<br>• 8.4: habit reversal	• Educational materials<br>• Group discussion<br>• Feedback from the leader and other peers	• We developed treatment content for teaching self-management behavior change skills<br>• We created the action plan form, the reflection form, and the long-term action plan form to identify the goal; review progress; and identify challenges, strategies, and resources to complete the goal behavior

Environmental context and resources	• 12.1: restructuring the physical environment<br>• 12.5: adding objects to the environment<br>• 12.2: restructuring the social environment<br>• 1.2: problem-solving<br>• 3.2: social support (practical)<br>• 7.1: prompts/cues	• Educational materials<br>• Community resources page<br>• Group discussion	• We developed treatment content for teaching skills to identify environmental barriers and resources<br>• We created a community resources page to identify local resources<br>• Participants shared experiences of their journey to address environmental or other contextual barriers

[a]BCTs were linked to MoAs. The links between BCTs and MoAs were identified by Carey et al [40], and BCTs were presented according to the BCT Taxonomy v1 developed by Michie et al [39].

[b]Practical applications implemented via SMS text messaging.
